# A Novel Microfluidic Point-of-Care Biosensor System on Printed Circuit Board for Cytokine Detection

**DOI:** 10.3390/s18114011

**Published:** 2018-11-17

**Authors:** Daniel Evans, Konstantinos I. Papadimitriou, Nikolaos Vasilakis, Panagiotis Pantelidis, Peter Kelleher, Hywel Morgan, Themistoklis Prodromakis

**Affiliations:** 1 Nanoelectronics & Nanotechnology Research Group, Electronics and Computer Science, University of Southampton, Southampton SO17 1BJ, UK; k.papadimitriou@ucl.ac.uk (K.I.P.); n.vasilakis@soton.ac.uk (N.V.); hm@ecs.soton.ac.uk (H.M.); 2 Centre for Immunology and Vaccinology, Division of Infectious Diseases, Department of Medicine, Imperial College London, London SW10 9NH, UK; panagiotis.pantelidis@nhs.net (P.P.); p.kelleher@imperial.ac.uk (P.K.); 3 Infection and Immunity, North West London Pathology, Imperial College NHS Trust, Charing Cross Hospital, London W6 8RF, UK; 4 Institute for Life Sciences, University of Southampton, Southampton SO17 1BJ, UK; 5 Zepler Institute for Photonics and Nanoelectronics, University of Southampton, Southampton SO17 1BJ, UK; t.prodromakis@soton.ac.uk

**Keywords:** cytokine detection, eELISA, lab-on-PCB, microfluidics, PCB biosensors, point-of-care diagnostics

## Abstract

Point of Care (PoC) diagnostics have been the subject of considerable research over the last few decades driven by the pressure to detect diseases quickly and effectively and reduce healthcare costs. Herein, we demonstrate a novel, fully integrated, microfluidic amperometric enzyme-linked immunosorbent assay (ELISA) prototype using a commercial interferon gamma release assay (IGRA) as a model antibody binding system. Microfluidic assay chemistry was engineered to take place on Au-plated electrodes within an assay cell on a printed circuit board (PCB)-based biosensor system. The assay cell is linked to an electrochemical reporter cell comprising microfluidic architecture, Au working and counter electrodes and a Ag/AgCl reference electrode, all manufactured exclusively via standard commercial PCB fabrication processes. Assay chemistry has been optimised for microfluidic diffusion kinetics to function under continual flow. We characterised the electrode integrity of the developed platforms with reference to biological sampling and buffer composition and subsequently we demonstrated concentration-dependent measurements of H_2_O_2_ depletion as resolved by existing FDA-validated ELISA kits. Finally, we validated the assay technology in both buffer and serum and demonstrate limits of detection comparable to high-end commercial systems with the addition of full microfluidic assay architecture capable of returning diagnostic analyses in approximately eight minutes.

## 1. Introduction

Biomarker analysis is one of the cornerstones of medical evaluation and PoC diagnostics have demonstrated the potential to become fundamental medical tools when rapid, sensitive, and user-friendly biomarker analysis in non-laboratory environments is required. Research into PoC devices has introduced a range of novel and optimised componentry featuring different microfluidic integration methodologies, new smart materials, novel data analytics and various connectivity systems [1,2,3,4,5,6,7,8,9,10,11,12,13,14,15,16,17]. However, such technologies are usually showcased in isolation under optimised laboratory conditions [18,19,20,21,22], and while the **A**ffordable, **S**ensitive, **S**pecific, **U**ser friendly, **R**obust and Rapid, **E**quipment-free, and **D**eliverable to end-users (“ASSURED”) criteria [23] proposed by WHO can be applied effectively to individual assay, reporter or detection components, they are most of the times less readily met by full PoC field tools.

For example, molecular components of assay technology are often developed for electrochemical reporter systems using expensive high quality electrodes able to function with a far higher sensitivity than, for example, the basic elemental surfaces we employ herein. This could result in a requirement for further assay optimisation in order to integrate with true PoC field systems. Similarly, electrochemical detection systems appropriate for PoC analysis are often developed using purified reporter reagents rather than fluids representative of true sample matrices, and under fluidic architecture favourable to electrode operation rather than assay or reporter kinetics. Furthermore, complete and functional systems are required to overcome resistance to uptake by end users, who must have complete confidence in their equipment. Therefore, the aim to develop kits capable of detecting and processing bio-information on-chip in the most cost- and space-effective manner still remains a challenge [9,24].

Many different methods and materials have been proposed, in order to tackle the ASSURED criteria bottleneck. Lateral flow assays [25,26,27,28,29] and smartphone-based [30] colorimetric [31,32,33] and electrochemical [34,35,36] PoC devices are only few, indicative examples of the vast work been done so far towards PoC testing devices that can reduce detection time, increase detection accuracy and ultimately reduce overall cost. Lab-on-PCB (LoPCB) is an alternative approach to PoC diagnostic systems that could reduce the costs associated with complex detection architectures [37,38,39,40,41,42,43,44]. By developing effective diagnostic systems utilising the already matured PCB technology and manufactured using standard materials and processes could further lower the cost of the PCB-based biosensing platforms, in line with ASSURED criteria, without sacrificing detection accuracy [45]. In mass production, the PCB-based sensors combined with bespoke PCB-based microfluidics could reach a fabrication cost close to the one of the undoubtedly cost-effective paper-based PoC devices (excluding the electronics unit and the chemical reagents). Moreover, a further advantage of this approach is the ability to integrate, if necessary, sample and reagent processing, assay chemistry, microfluidics, sensing architecture (e.g., electrochemical cells) and bespoke circuitry all at the surface of the PCB in a monolithic manner. Such integration inevitably favors the development of PoC platforms with smaller footprint and consequently development cost [46]. Combining both biochemistry and electronic biosensing on the same sensing platform may also reduce noise interference due to connectivity issues which could increase the signal-to-noise ratio (SNR) of the measurement [47]. 

In this work, we demonstrate a complete LoPCB biosensor platform with bespoke in-line assay cells, electrochemical cells and full microfluidic architecture that significantly builds on previous, proof-of-concept static LoPCB platforms [48,49,50,51]. While concepts and philosophies are, in general, consistent with the basic principles of LoPCB systems [37,38,46,49,50], the implemented system demonstrated herein utilises a novel system architecture and an entirely different electrochemical treatment, illustrating a system and electrochemical reaction tailored for ELISA protocols. The assay process is conducted under precisely controlled flow. Amperometric detection is performed using a bespoke, multi-channel, custom-made high-performance bioinstrumentation platform [50,52]. We exploit inexpensive substrates fabricated by our industrial partner (Newbury Electronics Ltd., Newbury, UK) using standard, mature commercial production techniques [46,53,54]. 

We demonstrate full assay chemistry, adapted from a commercial IGRA (R + D Systems, Minneapolis, MN, USA), occurring in bespoke microfluidic assay cells at an Au surface. The system is comprised of assay cells and electrochemical detection cells linked microfluidically. The target antigen is captured from the sample in concentration-dependant manner and subsequently horseradish peroxidase enzyme (HRP) is localised according to captured analyte concentration via a second primary antibody of alternative epitope. Reporter fluid 3,3′,5,5′-tetramethylbenzidine (TMB) is converted from substrate to product by the HRP at a rate determined by the concentration of localised HRP, and delivered to an integrated electrochemical cell. H_2_O_2_, as the enzyme co-factor, is consumed at a rate commensurate to TMB conversion. In the electrochemical cell amperometric analysis occurs at Au working and counter electrodes. We report system performance comparable to current industry standard colorimetric analyses and other indicative portable IFNγ detection systems.

The basic model assay is a commercially available IFNγ ELISA from R + D Biosystems, the only major adaptation being the expression of the primary capture antibody in cysteinylated ScFv format. IFNγ is a pro-inflammatory cytokine with a central role in innate and acquired immunity. In the clinical laboratory IGRAs are used routinely in at risk populations to diagnose latent TB infection (LTBI) [55]. The presented assay boards can process eight unique channels (while electronics kit can support up to 16 simultaneous measurements), allowing us to run eight distinct assays and appropriate controls at once on a single assay board, for demonstrating the parallel interrogation of full diagnostic suites of biomarkers in a single process.

## 2. Materials and Methods 

### 2.1. TMB Detection

TMB was prepared by adding one TMB tablet (T5525, Sigma Aldrich, St. Louis, MO, USA) to 10 mL deionised water and 5 µL 30% (by volume) H_2_O_2_. The reagent was centrifuged at 16,000 RCF for 30 min to remove particulates and decanted to a clean tube.

### 2.2. Assay-Incubated

Assays were performed in bespoke measurement cells at the PCB surface. Au assay surfaces were used to localise cysteinylated Fab’ antibodies via covalent thiol linkage. Fab’ antibodies were prepared at a concentration of 40 µg/mL in PBS and 100 µL incubated overnight in each assay cell at 4 °C. Assay wells were rinsed twice with PBS and 500 µL of 1% BSA (W:V) in PBS introduced to each well to block exposed hydrophobic surfaces. Blocking was allowed to progress for 2 h at room temperature (RT). Assay cells were rinsed twice with PBST (1 × PBS, 0.05% tween_20_-by volume) and titrated IFNγ flow was initiated. The IFNγ titration was prepared in log2 dilution in PBS across 8 assay points from a top concentration of 2 ng/mL. IFNγ samples were incubated at RT for 40 min. Cells were washed twice with PBST and once with PBS. Biotinylated detection antibody (R + D Biosystems) was prepared at 200 ng/mL in PBS and added to each cell. Incubations were allowed to progress for 40 min at RT. Cells were washed twice with PBST and once with PBS. Streptavidin-HRP was prepared in PBS at a dilution of 1:20 from the R + D Biosystems kit stock (no data provided for concentration). Streptavidin-HRP working dilution was added to each cell and incubated for 15 min at RT. Cells were washed twice with PBST and once with PBS. TMB substrate was prepared using stock tablets (T5525, Sigma-Aldrich) dissolved in 10 mL of deionised water containing 5 µL 30% (by volume) H_2_O_2_. TMB was flowed directly across the assay surface to the electrochemical detection cell, or incubated at the assay surface (as indicated in the text). Electrochemical measurements were taken in the electrochemical cell region of the assay board. Electrochemical cells consist of three Au surface electrodes and an Ag/AgCl reference electrode. Amperometric measurements were taken using the described in-house electronic control board providing a stable reference electrode bias of + 0.87 V. Colorimetric analysis was conducted using a GloMax spectrophotometer (Promega, Masison, WI, USA) recording at 450 nm using TMB reagent released though the waste port of the assay cell.

### 2.3. Assay in Plasma

The assays conducted under plasma sample matrix were performed using the same protocol applied to standard assays in PBS with the exception that IFNγ standards were diluted into plasma.

### 2.4. Assay Boards

Assay boards were fabricated through standard commercial PCB manufacture techniques. A 500 μm thickness flame retardant-class 4 (FR-4) laminate cladded on both sides with 35 μm thick copper layers was used for the two-layer PCB. The chemically etched copper structures on the PCB were gold plated in a second phase of the process. The solder mask layer on both sides of the boards assured the planarisation of the device to facilitate the PMMA-based in-house microfluidic integration. The gold plated assay surface was approximately 100 × 5 mm and the fluidic cell was approximately 100 µL in volume. The electrochemical cell consisted of three gold working electrodes, one Au counter electrode and a Ag/AgCl reference electrode. Cells were formed over the PCB surface using PMMA etched using a laser cutter (Epilog Helix, Epilog, UK) operating at 50% power and 60% speed, with two etching passes across the surface.

### 2.5. The 16-Channel Bio-Instrumentation Board

All electrochemical measurements have taken place using the 16-channel bioinstrumentation platform firstly shown in [50,52], where the technical characteristics of the instrument are mentioned in detail. The overall design of the board revolves around the idea of immediate digitisation of the sensitive analogue sensor signals [56], process them by means of standard digital signal processing techniques and subsequently present the results to the user in digital form, either to a PC or to the embedded on-board TFT touch screen. Due to its low power consumption, the instrument can be battery-operated, enhancing its portability inside and outside the laboratory environment.

## 3. Results and Discussion

**Figure 1:**
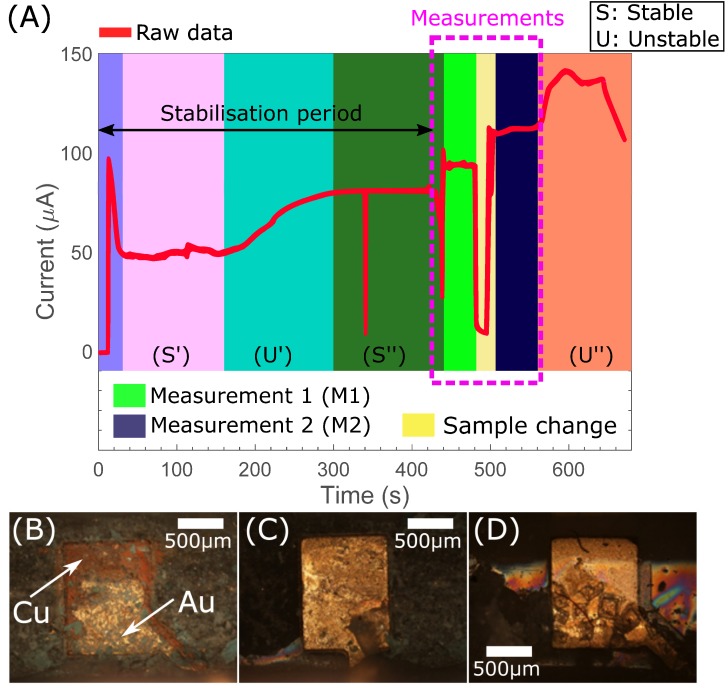
(**A**) Indicative raw data using a chloride ions-based buffer, detailing stable and unstable regions of sensor report. Two measurements are included (M1 & M2) to demonstrate the electrochemical capability of the system; (**B**–**D**) Images at ×30 magnification of working electrodes following electrochemical assessment of various chloride containing and non-chloride containing solutions; (**B**) Working electrode after measurement of chloride containing solution. Significant corrosion following extended electrochemistry with chloride ion containing solution; (**C**) Unused electrode from the same cell as (**B**) demonstrating that corrosion is an active process; (**D**) Working electrode from electrochemistry of non-chloride containing solution supports the chloride ion mediated corrosion hypothesis.

We require electrochemical detection using components manufactured by standard commercial processes, thus, our electrode compositions and structures are considerably less refined than those employed in general electrochemical practice and represent a novel step in the development of PCB-based electrochemical reporter systems. In consideration of the practicability of our electrochemical reporter systems in terms of material and subsequent signal stability, we have initially investigated the effect of two different types of electrochemical buffers upon our system’s performance. The first type of electrochemical buffer included chloride ions (PBS), while in the second one chloride compounds were excluded (HEPES). Figure 1A shows an indicative data trace (amperometric signal recorded by our in-house electronics) from a gold plated PCB-based sensing pad, generated by our bespoke graphical user interface (GUI). In this case, an electrochemical buffer that include chloride ions has been employed, leading to the high-current response shown in Figure 1A. More specifically, in phase 1 (S’) the signal is stable because the thin gold electrode surface has yet to corrode through to the underlying copper primer. In phase 2 (U’) the gold electrode surface is actively corroding as a secondary electrochemical cell is set up at the exposed interface of the Au electrode surface and the copper primer. In phase 3 (S’’) sufficient surface area of copper primer has been exposed that electrochemical activity associated with the copper surface is now interacting with the buffer rather than the remaining gold electrode. While manually controlling the fluidic, assay, and electrochemical reporter processes stability deviations were easily observed and avoided, these advantages are not available in a fully automated system.

Figure 1B,D compare microscopy of electrodes that have demonstrated the high current phase (Figure 1B) with chloride ions in the buffer (PBS) to that of electrodes that have only been used with HEPES buffer (Figure 1D). Corrosion is clearly visible in the first case whereas we see only dehydrated buffer crystals in the second. The inclusion of a chloride ion and an oxidation source has been shown to lead to the reduction and resulting dissolution of gold surfaces [57]. Consideration of relevant reduction potentials demonstrates that if Au and Cu are electrically connected and exposed to electrolyte they can form a secondary electrochemical system with reduction occurring at the Au surface while Cu is oxidised. We observe damage to the Au electrode surface and under microscopic analysis we reveal exposed copper (Figure 1B).

Both gold and copper are able to form compounds that appear green in colour, gold(IV) chloride and copper(I) chloride (white solid) appears green when contaminated with small amounts of the light brown copper(II) chloride [57]. We assessed solubility and demonstrated minimal dissolution of the green compound in water, and following addition of iron (II) oxide we failed to generate a precipitate. Gold chloride is more readily soluble in water than copper chloride, furthermore a solution of gold chloride will precipitate elemental gold on addition of iron (II) oxide while copper chloride will not [57]. Either situation would indicate dissolution of gold at the electrode surface. Our results and observations are consistent with an active process dissolving the gold electrode surface, as unconnected working electrodes in an active cell do not corrode (Figure 1C).

### 3.1. Demonstration of Electrochemical H_2_O_2_ Measurement

**Figure 2:**
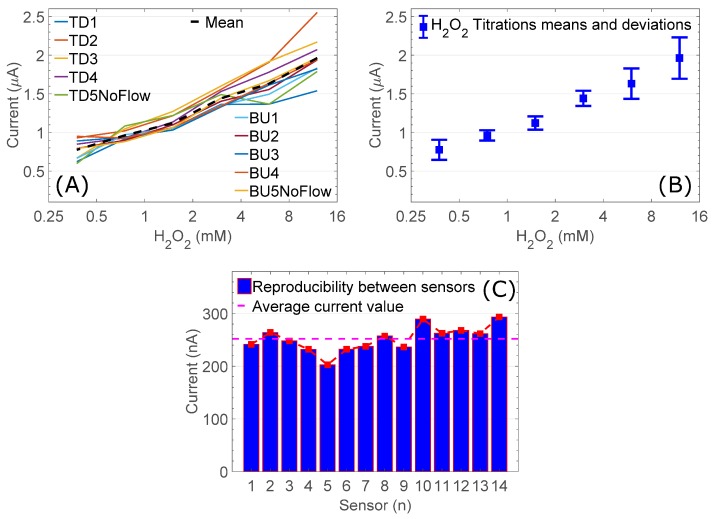
(**A**,**B**) Reproducibility within sensor. Ten H_2_O_2_ titrations were assessed independently on a single sensor to establish the reproducibility of the electrochemical measurement scheme. (**A**) Collected results; (**B**) their mean values and standard deviations; (**C**) Reproducibility between sensors. Electrochemical reproducibility is demonstrated between sensors by analysing a single H_2_O_2_/HEPES in 14 different electrochemical cells. Between sensor deviations could be eliminated through calibration to a single sample in the future (TD: Top-Down, BU: Bottom-Up).

Figure 1 demonstrates that the use of non-chloride containing solutions, should allow for a more reliable measurement system, operating at lower current ranges. Some variability due to noise is inevitable in a solid-liquid interface electrochemical cell especially when molecular surface interaction times have been reduced to establish optimal practical functionality. We calculated mean values within each 100 measurement counts across areas of variable noise and we found these averages to be highly consistent thus we conclude that the observed noise does not affect accuracy in measurements. Reproducibility is demonstrated by the generation of numerous H_2_O_2_ concentration measurement curves. Ten curves are shown in Figure 2A,B detailing H_2_O_2_ concentrations between 0.375 and 12 mM and associated electrochemical measurements. The obtained results are consistent and reproducible in the 0–4 mM H_2_O_2_ concentration range, while at higher molarities the signal becomes marginally more variable. The delivered range for the commercial standard TMB colorimetric assay begins with H_2_O_2_ at approximately 6.4 mM, as defined by H_2_O_2_ concentrations in standard phosphate citrate buffer tablets (Cat#79379, Sigma-Aldrich). We conclude that our system will provide a working assay for H_2_O_2_ without altering concentrations in current industry standard colorimetric assay protocols.

We also assessed between sensor reproducibility by applying a single H_2_O_2_ sample set to fourteen individual sensors. We demonstrate a co-efficient of variation of 0.095, which corresponds to approximately 10% variability (Figure 2C). We are able to calibrate signals from multiple sensors by analysis of a fixed concentration calibration reagent and adjust our numerical output accordingly. In a fully commercial device this could occur at the software level and the device will output values following internal calibration, which is a standard procedure in the majority of laboratory and field sensing devices. The described measurements were taken using 10 mM HEPES containing various concentrations of H_2_O_2_. As H_2_O_2_ is the only variable we conclude that these measurements are characteristic of our desired PoC sensor output and allow us to loosely predict the values or value patterns that should be returned when measuring H_2_O_2_ in other solute matrices. We use this performance pattern as a means to compare and characterise performance in other, more complex solute environments.

### 3.2. TMB Measurement Protocol Is Determined by H_2_O_2_ Depletion Kinetics

**Figure 3:**
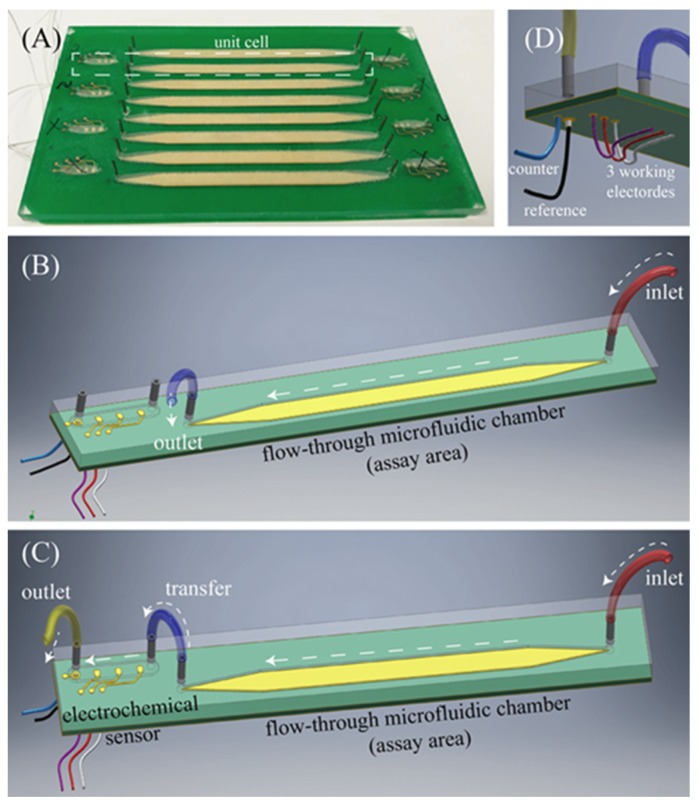
(**A**) Full PCB assay and electrochemistry board showing 8 independent assay areas (long gold strips) and 8 independent electrochemical cells (dashed line highlights a single unit cell); (**B**) A three-dimensional graphical representation of the unit cell fluidic arrangement during the initial phase of the assay; (**C**) A three-dimensional graphical representation of the unit cell fluidic arrangement during the second phase of the assay, detailing fluidic ports and connections between assay area and electrochemical sensor; (**D**) Detail of the biosensor’s electrical connections at the bottom of the PCB.

Our full in-line assay system consists of an assay region and electrochemical cell at the surface of a bespoke PCB, shown in Figure 3. The assay area consists of a strip of deposited gold enclosed beneath a PMMA cover, laser etched to create a microfluidic channel across the gold surface. Microfluidic ports allow sample inflow and egress. During the assay phase sample is moved out of the assay area to a waste channel (see Figure 3B). During the TMB reagent application step the channel is switched to allow flow into an electrochemical cell consisting of Ag/AgCl reference electrode, Au counter electrode, and three Au working electrodes that can operate either together or independently depending on connections (as shown in Figure 3C).

**Figure 4:**
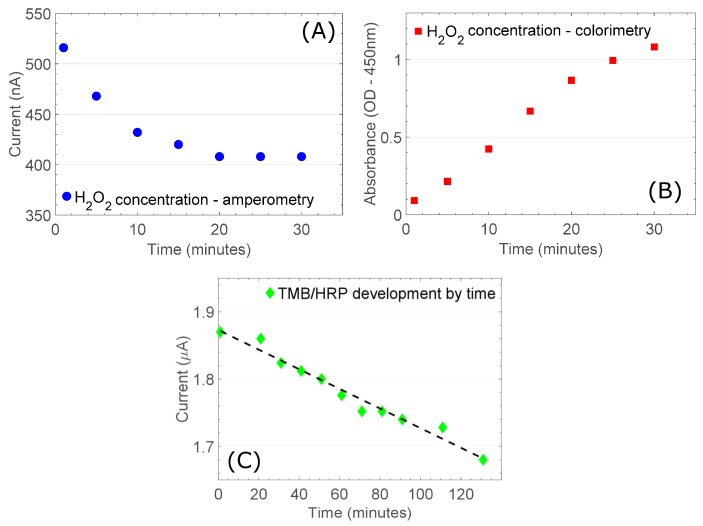
H_2_O_2_ concentrations are measured by (**A**) amperometry and (**B**) colorimetry; (**C**) TMB conversion by low enzyme concentrations over an extended time period demonstrates a high level of sensitivity in the electrochemical detection technique.

Figure 4 shows amperometric assessment of H_2_O_2_ compared to colorimetric assessment of TMB product. These analyses were performed on the same samples (following addition of 0.1 × Vol. 1 M H_3_PO_4_). Standard product concentrations were defined by reaction time. During the reporter phase of the assay H_2_O_2_ is a required co-factor in the conversion of TMB by the HRP enzyme. As such, H_2_O_2_ depletion can be used to determine HRP concentration. In the standard IFNγ release ELISA quantitation is performed colorimetrically as determined by concentration of TMB product, which is established according to HRP concentration. Therefore, IFNγ concentration is also measurable through H_2_O_2_ quantitation.

More specifically, in Figure 4A,B we show how changes in colorimetric signal correspond to changes in H_2_O_2_ concentration using our amperometric detection system. Our results confirm that HRP concentration may be quantified both by progressive H_2_O_2_ depletion over time, and by initial H_2_O_2_ depletion. To demonstrate consistency in H_2_O_2_ depletion as TMB substrate conversion progresses we produced a similar series of measurements detailing H_2_O_2_ depletion in the presence of a very low concentration of HRP enzyme (Figure 4C).

Amperometric H_2_O_2_ measurements reach saturation when all available H_2_O_2_ is depleted, after approximately 20 min (Figure 4A), while TMB product saturation only starts to become evident at 30 min (Figure 4B). As H_2_O_2_ is a required co-factor for TMB conversion its depletion from the electrolyte will be evident before the appearance of the associated TMB product. Therefore, rather than allowing static incubation in an assay chamber we used a continuous flow-through arrangement in a microfluidic assay chamber. This is beneficial both in light of the observations we have made concerning H_2_O_2_ depletion kinetics and with respect to device functionality. The new system allows us to flow reporter reagents directly through the assay chamber to the electrochemical detection cell, eliminating the need for complex and laborious time-consistency steps.

To accurately quantify H_2_O_2_, measurement must be optimised in accordance with enzyme H_2_O_2_ uptake kinetics. To measure the rate of depletion of H_2_O_2_ in solution we can incubate a TMB/H_2_O_2_ reagent sample in an assay well for a certain period of time. However, in colorimetric measurements it is possible to stop the samples from further enzymatic conversion by rapidly reducing pH. This is not an option in electrochemical assessment as the added acid provides an electrochemical signal that dwarfs that of H_2_O_2_ at relevant concentrations, thus, obscuring accurate quantification. This means that added precautions must be taken to ensure that all samples develop for precisely the same amount of time in order for them to be quantitatively comparable. For this reason, we use immediate H_2_O_2_ uptake to assess HRP concentrations.

Continual reagent flow minimises the possibility of unequal reagent to assay surface contact times and therefore provides the most accurate quantification. However, static incubation of TMB reagent at the assay surface would effectively amplify differences in H_2_O_2_ consumption due to increased reaction times, therefore, improving the precision of our assay system. Either system is practicable using the same device architecture.

The timing of TMB reagent contact with the assay surface via flow rate provides an assay optimisation scheme that increases the versatility of our device for the inclusion of different antibody-antigen pairs. This is a highly significant factor for future engineering as the system is intended for rapid multiplexed analysis of entire diagnostic biomarker suites by splitting a single inducted sample into multiple flow channels and thus multiple assay environments. We further demonstrate a very detailed signal resolution confirming adequate system sensitivity. By applying a very low HRP concentration we stretched the reaction across more than 2 h and found returned amperometric values remained consistent with the faster reactions we have shown (Figure 4C).

### 3.3. Prototype IFNγ PoC Detection Assay Demonstration

**Figure 5:**
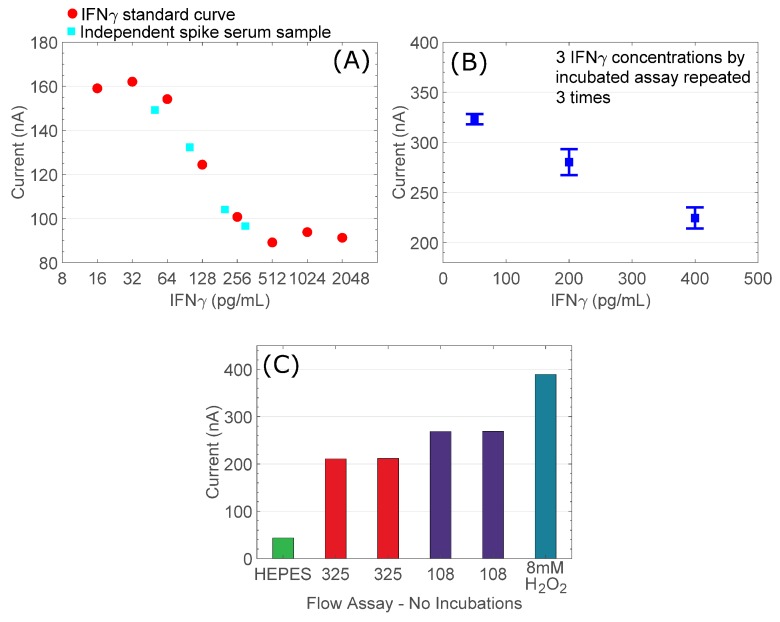
(**A**) Full IFNγ assay data generated using bespoke assay areas at the PCB surface (red circle), showing assay points from samples of IFNγ spiked into normal blood plasma (cyan square); (**B**) Three concentrations of IFNγ, each repeated three times; (**C**) IFNγ concentrations assessed by full flow assay protocol. All assay reagents are supplied under flow without any static incubation steps.

TMB reagent additions were performed through a microfluidic flow cell, flowing TMB substrate solution continuously over the assay surface and directly into the electrochemical measurement cell without arresting delivery. Figure 5A shows a curve generated following full IFNγ assay chemistry on a gold assay surface, detailing IFNγ concentrations titrated from 16–2048 pg/mL. Using the same assay protocol, we show three repetitions of three samples of different IFNγ concentration measured using the same sensor (Figure 5B). Given negligible demonstrated within sensor variabilities, this experiment provides a measure of reproducibility of assay chemistry within our prototype system. We show acceptable sample grouping and standard error. We also ran a number of samples in plasma matrix, which show consistency with the analyte-spiked buffer titrations demonstrating that the system is functional using the required medical sample matrix. 

Finally, we show an automated assay performed under microfluidic flow. User input, consisting of changing sample syringes and starting/stopping flow, was confined to operation of a syringe pump and switching between waste and electrochemical cell outputs from the assay cell. Due to limited numbers of sensor and assay boards we were not able to produce numerous repetitions of the following quantifications. However, the reproducibility and statistical variance of each component of the system has been investigated thoroughly. The following *end to end* assay runs demonstrate the capability of the automation scheme we have employed and the potential to significantly reduce assay times to PoC-relevant periods. 

We show excellent reproducibility of repeated analyses of IFNγ samples at 325 and 108 pg/mL, and these samples fall neatly between positive and negative controls representing 10 mM HEPES (negative control) and fresh TMB reagent containing 8 mM H_2_O_2_ (Figure 5C).

Having defined the working parameters of a full in-line assay and reporter system we generated data to demonstrate the functionality of a complete end to end flow-through assay prototype. We show an adequate correlation between amperometric signal and supplied IFNγ concentration (in log2 titration series) within a full sigmoid curve showing signal extinction at low concentrations, a linear response region, and signal saturation at high concentration. Our limit of detection (LoD), conservatively approximated at 40 pg/mL, is broadly similar to the published R + D systems colorimetric limit of detection (15 pg/mL) given the full range of the assay (15–2000 pg/mL) and our own analysis of the R + D systems kit (24 pg/mL), and falls cleanly within the medically relevant IFNγ range in TB diagnosis by IGRA. We further confirmed the accuracy of our results by running IFNγ spiked plasma samples at four distinct concentrations and comparing them to our curve. Estimates of concentration are approximately 93–97% accurate (see Table 1). This analysis shows that our in-line assay prototype is functional and appropriate to purpose with respect to IFNγ quantitation in blood plasma for clinical TB diagnosis by IGRA.

Variability is derived from additive effects of mechanical handling, assay chemistry, electrochemical analysis, including different assay surfaces and different electrochemical sensors. Essentially this analysis represents the reproducibility of the full prototype system. Our results show good grouping appropriate for IFNγ quantitation. A final working device will eliminate the largest source of variability (between sensors) through device calibration. Values are shown in Table 2.

The device design exploits microfluidic technology to eliminate molecular diffusion requirements and dramatically reduce assay incubation times. Our final analysis once again uses identical assay component concentrations but we have completely eliminated the incubation steps. Our assay required ~3 min for analyte (IFNγ) flow (100 µL/min), ~3 min for Streptavidin-HRP flow (100 µL/min), and ~2 min for TMB flow (100 µL/min). Electrochemical readings must be taken immediately due to enzyme H_2_O_2_ uptake kinetics described above. Discounting minimal handling times, the entire PoC assay was therefore achieved in roughly 8 min. Finally, Table 3 summarises our system’s quantitative performance and compares it to other, indicative, state-of-the-art IFNγ detection systems, each one exploiting a different detection method and fabrication materials.

## 4. Conclusions

We demonstrated an integrated biosensor system capable of detection of IFNγ for use in standard TB diagnosis by IFNγ release assay for PoC purposes. This is only one application as our innovations apply primarily to reporter compound detection (TMB/H_2_O_2_), thus, with suitable assay conversion can be applied to the majority of current TMB-based colorimetric analyses. We have demonstrated performance and variability within each system component to include measurement of TMB conversion by HRP via H_2_O_2_ depletion and antibody binding-based detection and measurement of IFNγ. Furthermore, we have investigated within and between sensor variations and demonstrate these to be comparable to traditional commercial systems. Finally, we have shown the working prototype, reporting limits of detection and precision comparable to current industry standard analyses and other indicative portable IFNγ detection systems and we show the entire assay process is possible within eight min. Future work will revolve around extensive testing, in confirmation of results herein with statistically significant sample population sizes and for analysis of real clinical samples to be demonstrated in statistically significant numbers.

## Figures and Tables

**Table 1:** Assayed IFNγ spiked serum samples were compared to a calibration curve from IFNγ titrated in buffer to indicate the accuracy of clinical measurements. Percentage difference using the fit equation: $f\left(x\right)=\frac{a}{\left(1+\exp\left(-b\;x\right)+c\right)}+c$ ranges between 3–7%.

Sample (pg/mL)	Predicted Value (nA)	Measured Value (nA)	|%Difference|
50	157.610	149.260	5.300
100	141.560	132.340	6.510
200	97.370	104.120	6.920
300	93.680	96.620	3.130
**Mean difference:** 5.465%			

**Table 2:** Three repeats of each of three sample concentrations were measured using the prototype assay system to demonstrate the combined variability of the full assay system.

Repeat	50 pg/mL	200 pg/mL	400 pg/mL
1	0.326	0.264	0.222
2	0.325	0.294	0.236
3	0.317	0.277	0.215
mean	0.323	0.278	0.224
Standard deviation	0.005	0.015	0.010
Coefficient of variation	0.015	0.054	0.045

**Table 3:** Indicative, state-of-the-art IFNγ-detection systems, utilising different methods and materials.

Ref.	Detection Limit (pg/mL)	Detection Range (pg/mL)	Method/Materials
[58]	3.4	5–1000	Impedance immunosensor/Paper-based.
[59]	0.4	0.4–40	Square wave anodic stripping voltammetry/MNPs & AuNPs.
[60]	0.048	0.1–10,000	PDDA/AuNPs.
[61]	1.3	1.3–210	Amperometry/GO & structure-switching aptamers.
[62]	520	1000–5000	Label-free EIS/Au IDE.
This work	40	16–2048	Amperometry/PCB.

## Data Access Statement

The data from this paper can be obtained from University of Southampton Institutional Repository (https://doi.org/10.5258/SOTON/D0715).
